# A Model of Motion Processing in the Visual Cortex Using Neural Field With Asymmetric Hebbian Learning

**DOI:** 10.3389/fnins.2019.00067

**Published:** 2019-02-12

**Authors:** Anila Gundavarapu, V. Srinivasa Chakravarthy, Karthik Soman

**Affiliations:** ^1^Department of Biotechnology, Bhupat and Jyoti Mehta School of Biosciences, Indian Institute of Technology Madras, Chennai, India; ^2^Department of Bioengineering, University of California, Berkeley, Berkeley, CA, United States

**Keywords:** neural field models, weight asymmetry, pattern selectivity, lateral interactions, primary visual area (V1), middle temporal area (MT), medial superior temporal area (MST)

## Abstract

Neurons in the dorsal pathway of the visual cortex are thought to be involved in motion processing. The first site of motion processing is the primary visual cortex (V1), encoding the direction of motion in local receptive fields, with higher order motion processing happening in the middle temporal area (MT). Complex motion properties like optic flow are processed in higher cortical areas of the Medial Superior Temporal area (MST). In this study, a hierarchical neural field network model of motion processing is presented. The model architecture has an input layer followed by either one or cascade of two neural fields (NF): the first of these, NF1, represents V1, while the second, NF2, represents MT. A special feature of the model is that lateral connections used in the neural fields are trained by asymmetric Hebbian learning, imparting to the neural field the ability to process sequential information in motion stimuli. The model was trained using various traditional moving patterns such as bars, squares, gratings, plaids, and random dot stimulus. In the case of bar stimuli, the model had only a single NF, the neurons of which developed a direction map of the moving bar stimuli. Training a network with two NFs on moving square and moving plaids stimuli, we show that, while the neurons in NF1 respond to the direction of the component (such as gratings and edges) motion, the neurons in NF2 (analogous to MT) responding to the direction of the pattern (plaids, square object) motion. In the third study, a network with 2 NFs was simulated using random dot stimuli (RDS) with translational motion, and show that the NF2 neurons can encode the direction of the concurrent dot motion (also called translational flow motion), independent of the dot configuration. This translational RDS flow motion is decoded by a simple perceptron network (a layer above NF2) with an accuracy of 100% on train set and 90% on the test set, thereby demonstrating that the proposed network can generalize to new dot configurations. Also, the response properties of the model on different input stimuli closely resembled many of the known features of the neurons found in electrophysiological studies.

## Introduction

Visual motion is experienced by living organisms either due to self-motion with respect to the environment or by the motion of individual objects in the environment. Nearly half a century of research has provided a detailed description of motion processing in mammalian visual cortex. For example, we know that motion is processed along the visual motion pathway that consists of at least three hierarchical cortical stages—primary visual cortex (V1), middle temporal area (MT), and medial superior temporal area (MST) (Adelson and Movshon, 1982; Movshon et al., 1985; Movshon and Newsome, 1996; Pack et al., 2001; Orban, 2008; Gilaie-Dotan, 2016). Neurons in each of these stages have diverse response properties and are involved in different aspects of motion processing.

The first cortical stage of primate motion processing starts at V1 where a subset of cells is highly direction selective (Hubel and Wiesel, 1968; Movshon and Newsome, 1996). These cells have relatively small spatiotemporal receptive fields (Hubel and Wiesel, 1974) and encode the direction of motion of local features. These motion cues are often different from the motion of the visual pattern; hence locally encoded motion cues are ambiguous (Wallach, 1976) and result in the so-called *aperture problem* (Fennema and Thompson, 1979; Wuerger et al., 1996; Pack et al., 2001, 2003). These local motion cues are integrated by the second stage cells at MT (Adelson and Movshon, 1982; Pack et al., 2001; Born and Bradley, 2005) that have relatively larger receptive fields and compute the direction of pattern motion. Earlier experimental studies of pattern motion selectivity were conducted with stimuli consisting of moving plaids (Rodman and Albright, 1989). They showed that MT cells are capable of encoding two-dimensional motion (pattern motion) while V1 cells encode one dimension of stimulus motion (component motion: the motion of a pattern boundary segment such as bar, edge and sinusoidal grating). MT is also thought to estimate overall pattern velocity by combining local velocity cues from V1 (Adelson and Movshon, 1982; Bowns, 1996, 2018). However, some cells in MT (Majaj et al., 2007) selective to components moving in preferred direction rather than the direction of pattern motion. From optical imaging and single-cell recording studies we know that MST cells receive projections from MT, and respond selectively to the higher order optic flow motion, including translation, radial, rotation and combinations of the latter two (Tanaka and Saito, 1989; Duffy and Wurtz, 1991; Orban et al., 1995; Morrone et al., 2000).

Efforts to model the properties of neurons in the motion pathway had progressed with the accumulation of physiological results. There are models that successfully account for various properties of V1 cells, such as orientation selectivity, ocular dominance, and direction selectivity. Adelson and Bergen (1985) used phase independent spatiotemporal filters (created using oriented Gabor functions) to achieve direction selectivity. The filters were designed as quadrature pairs tuned for both directions. Saul and Humphrey (1990) achieved direction selectivity by designing both lagged and non-lagged cells. A model of Simoncelli and Heeger (1998) demonstrated direction selectivity of V1 cells and pattern selectivity of MT cells by integration of constraints. The Heeger model is non-linear and simulated the moving stimulus-response as the sum of the responses to a set of sequential stimuli evenly spaced in time, with an explicit time variable. Others showed that activity-dependent self-organization results in direction selectivity (Shouno and Kurata, 2001; Miikkulainen et al., 2006). Miikkulainen et al. used intra-cortical circuitry to incorporate excitatory and inhibitory effects along with LGN lagged cells to achieve direction selectivity (Miikkulainen et al., 2006).

These early studies either processed the entire stimulus trajectory, or a subset of the trajectory via time-lagged input at a single time step, which is biologically unrealistic. Some models (Somers et al., 1995) focus on explaining a single functional property like orientation selectivity or direction selectivity, therefore accounting only for a subset of visual neural behaviors. The models proposed by Miikkulainen et al. (2006) attempt to explain diverse properties such as orientation selectivity, direction selectivity and ocular dominance of neuronal population in the Primary visual cortex which is the first stage in the motion pathway. Bichler et al. proposed an interesting 2 layer feedforward fully connected neural network model that can learn temporally correlated features directly from vision sensor data using biologically plausible unsupervised STDP learning scheme (Bichler et al., 2012). The biologically plausible motion estimation model (Bowns, 2018) which is an enhanced version of Component-Level Feature Model (Bowns, 2011), can estimate the motion trajectories successfully from 7,000 synthetic moving images.

In this paper, we describe a computational model that can explain the diverse properties of the neurons, such as direction selectivity, pattern selectivity, and translation flow selectivity at different regions of the motion pathway. The proposed network can develop Gabor like receptive fields (Marcelja, 1980; Bowns, 2018) as a result of training the weight connections with moving bars using biologically plausible unsupervised learning rule. A study (Fu, 2004) reported that visual response properties like orientation selectivity, direction selectivity etc. are crucially dependent on the lateral interactions in the visual cortical circuit. They hypothesized that during adaptation Spike-Time-Dependent Plasticity (STDP) allows motion stimuli to induce asymmetry in the intracortical connections. The crucial role of lateral interactions in the development of the retinotopic map (Philips and Chakravarthy, 2015) was recently modeled using LISSOM (Philips and Chakravarthy, 2015) which can be considered as a neural field model with short-range excitation and long-range inhibition. Thus, each neural field unit has excitatory lateral connections with its neighboring units and inhibitory lateral connections with units farther away. We take our lead from this model and used asymmetric Hebb rule to introduce asymmetry in the intra-cortical circuit during adaptation to visual motion stimuli. The famous Hebb postulate (Morris, 1999) can be described as follows:

When an axon of cell A is near enough to excite cell B or repeatedly or persistently takes part in firing it, some growth process or metabolic change takes place in one or both cells such that A's efficiency, as one of the cells firing B, is increased.

## Model Architecture

**Figure 1:**
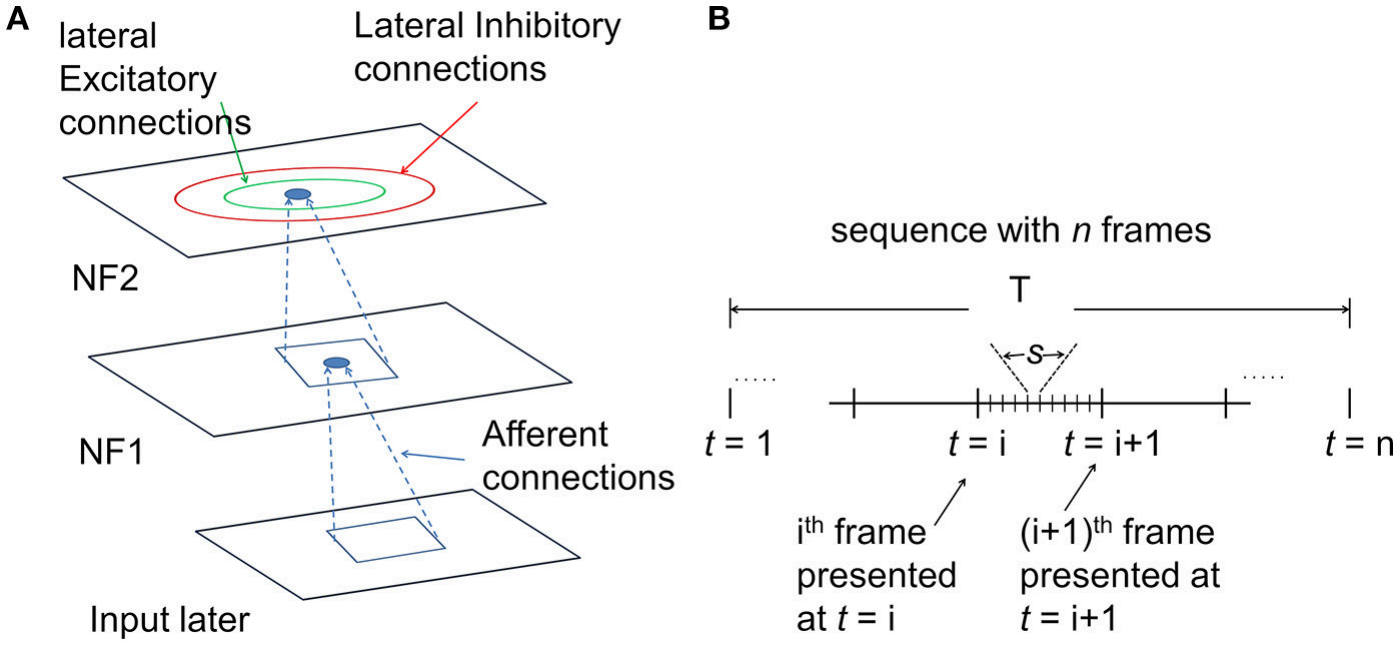
The architecture of the motion processing system. **(A)** *Neural field Model:* It consists of two NFs, analogous to V1 and MT of the visual cortex. Input layer represents the receptor surface such as the retina. Each NF is organized as a two-dimensional array of neurons with lateral connections. Every neuron has excitatory afferent (incoming; shown in dotted lines) connections from units in their square-shaped RF. Neighboring neurons have overlapping RFs. In addition, every neuron receives inputs from two types of lateral connections: excitatory connections (green circle represents excitatory radius) with nearby neighbors and inhibitory with neurons farther away (red circle represents inhibitory radius). **(B)** *the timeline of input sequence presentation to the network*: The model response to a moving stimulus was simulated at two different time scales. The sequence of *n* frames was presented to the network over a period of time *T*. Motion within the stimulus sequence was generated at several discrete time steps “t.” The number of time steps is equal to the number of frames within the sequence. For a given time “*t*” the lateral interactions were allowed to proceed for several time steps “*s*,” called the *settling time*.

The architecture of the proposed hierarchical motion processing network with two NFs is shown in Figure 1A. As described in Figure 1A every neuron makes lateral connections with neurons in its neighborhood in two ways: (i) short-range lateral excitatory connections and (ii) long-range lateral inhibitory connections. These lateral connections are permitted to be asymmetric. Also, every neuron is connected to its receptive field via afferent connections. All afferent and lateral connections are randomly initialized.

### Training Procedure

A number of simulations were conducted using traditional patterns used in earlier studies (Simoncelli and Heeger, 1998; Bowns, 2018) such as moving bars, moving plaids, moving RDS etc. to ensure the role of asymmetric lateral interactions in driving motion selective responses. To begin with, various network properties (number of NFs, NF dimension, and number of iterations in settling process) and parameters (receptive field size, excitatory and inhibitory radius, learning rates used during weight adaptation, scaling factors used in lateral interaction) need to be defined depending on the cortical region intended to model. For example, direction selectivity of V1 cells was modeled by a single NF, whereas the pattern selectivity of MT cells was modeled with a network of two NFs. The general strategy adopted for choosing the model parameters is discussed in the subsequent sections. In each simulation, for each NF, parameter set varies (as shown in Table 1) and is determined through trail-and-error method.

**Table 1:** Parameters used in various simulations.

**Parameter**	**Direction selectivity**	**Component and Pattern motion selectivity**				**Translational flow selectivity**	
	**Thin bar**	**Gratings and Plaids**		**Square object**		**RDS Translate**	
	**NF1**	**NF1**	**NF2**	**NF1**	**NF2**	**NF1**	**NF2**
Dim	20 × 20	20 × 20	13 × 13	13 × 13	15 × 15	29 × 29	22 × 22
RF	64 × 64	24 × 24	8 × 8	12 × 12	13 × 13	4 × 4	8 × 8
r_exc_	3	2	3	3	3	3	3
r_inhb_	10	10	4	6	7	4	5
γ_aff_	1	1	1	1	1	0.3	1
γ_exc_	3.9	8.2	4.8	2.8	2.2	0.68	15.68
γ_inhb_	1	1	3	1	1.5	1	1
α_aff_	0.05	0.05	0.05	0.3	0.3	0.05	0.05
α_exc_	0.05	0.05	0.05	0.3	0.3	0.05	0.05
α_inhb_	0.05	0.05	0.05	0.3	0.3	0.05	0.05
Ts	10	10	8	10	10	10	10
e	500	1,500	500	500	500	200	200
Image size	64 × 64	64 × 64	64 × 64	64 × 64	64 × 64	16 × 16	16 × 16

The training set is created with short sequences/videos, each composed of 10 images/frames at the most. During training, the individual sequence from the training set was drawn randomly and presented to the network image by image over the time period T (Figure 1B) so that, at a given time step *t*, the network has access only to the current image.

Each neuron in a given NF at time *t* first calculates its instantaneous afferent response, which is further modified by neighboring neurons through lateral interactions that result in a stabilized activity pattern. For a given time “*t*” the lateral interactions were allowed to proceed for several time steps “*s*,” called the *settling time*. Once the settled activity is obtained in the NF, the weights (both afferent and lateral) will get updated through asymmetric Hebbian learning (see the following section for details). Now the network is ready for the presentation of the next image at time *t*+*1*. This process is repeated until we present the last image of the sequence. Before presenting the next sequence, the neuron activity in the NF was reset to zero, bringing the neurons to the resting state. Presenting the entire training set once to the network is termed as an epoch. Training was carried out until the weights are saturated. Weights are called saturated if 80% of the change in weights (ΔW) approaches to 0. Once the training is completed, the network response was abstracted as a map (using the procedure described in the following sections) to check for the topographic self-organization. Also, the model results were compared with motion sensitivity results from electrophysiological experiments. All simulations were carried out using MATLAB.

### Equations Used for Training

#### Initial Response

For each image presentation, the initial activity *S*_*ij*_ of the neuron at *(i,j)* is computed as a scalar product of afferent weight vector *W*_*ij*_ and its receptive field *X*_*ij*_ Equation (1); σ is piecewise linear sigmoid activation function; γ_aff_ is a constant scaling factor.

(1) $$\begin{array}{r} S_{ij}=\sigma \left(\gamma_{aff}*\left(W_{ij}.X_{ij}\right)\right) \end{array}$$

As the afferent connections are random initially, the initial activity pattern on the NF was widespread and distributed all over the NF. This distributed activity was focused into a localized response by the effect of lateral interactions as follows.

#### Lateral Interactions

Each neuron's initial response was strengthened and sharpened by both short-range lateral excitation and long-range lateral inhibition over several time steps (Figure 1B). A number of time steps are represented by a parameter called the settling time (Table 1). At each of these discrete time steps “*s*,” the neuron combines its afferent stimulation with lateral interactions (Equation 2). During the iterations, the initial activity pattern that spreads over the substantial part of the NF was slowly converged into a focused patch of activity bubble and settles in the best responding area of the NF. Note that while the NF response settles down, the afferent input remains constant. The overall response of a neuron that combines both afferent and lateral interactions is described by the following equation.

(2) $$\begin{array}{l} \eta_{ij}\left(s\right)=\sigma (S_{ij}+\gamma_{exc}\sum_{kl}\eta_{ij}{\left(s-1\right)}^{*}E_{ij,kl}\\ \;-\gamma_{inhb}\sum_{kl}\eta_{ij}{\left(s-1\right)}^{*}I_{ij,kl}) \end{array}$$

where η_*ij*_ stands for the activity of the neuron at *(i,j), E*_*ij,kl*_, and *I*_*ij,kl*_ are excitatory and inhibitory weights from the neuron *(k,l)* to *(i,j)*. The relative strengths of excitatory and inhibitory lateral connections of each NF can be represented by constant scaling factors γ_exc_ and γ_inhb_.

#### Weight Adaptation

Once the activity has settled, both afferent and lateral weights for each neuron were modified. The afferent weight connection between NF unit *(i,j)* and input pixel *(k,l)* is modified as

(3) $$\begin{array}{r} \Delta W_{ij,kl}\left(t\right)=\;\alpha_{aff}*X_{kl}\;*\eta_{ij}\left(t\right) \end{array}$$

The lateral weights are modified according to a variation of the Hebbian learning. Classical Hebbian learning is temporally symmetric: weight update is dependent on the correlation between pre- and post-synaptic activity. We employ an asymmetric Hebbian rule (Schulz and Reggia, 2004) where the change in weight connection ΔW_ij,kl_ from *(k,l)* to *(i,j)* is computed as a dot product of pre- and post-synaptic neuron activities at different time steps as shown in Equation (4). Presynaptic activity is the settled activity of *(k,l)* for the previous frame η _kl_(t-1) and postsynaptic activity is the increase in the settled activity of *(i,j)* for the current frame η _ij_(t) relatively to the previous frame. The asymmetric Hebbian rule is combined with postsynaptic divisive normalization (Turrigiano, 1999) [Equation (5)] to prevent weights from increasing without bounds. The calculated new weight is used until the end of the next settling process.

(4) $$\Delta W_{ij,kl}(t)\;=\alpha *\max(0,(\eta_{ij}\left(t\right)-\eta_{ij}(t-1)))*\eta_{kl}(t-1)$$

where α is the parameter determining the rate of learning. For each type of connection (excitatory, inhibitory) separate learning rates were used.

(5) $$\begin{array}{r} W_{ij,kl}^{new}=\;\frac{W_{ij,kl}^{old}+\Delta W_{ij,kl}}{\sum_{u}\left(W_{ij,kl}^{old}\;+\Delta W_{ij,kl}\right)} \end{array}$$

where $W_{ij,kl}^{new}$ is new weight connection from neuron *(k,l)* to neuron *(i,j)* at each “*t*.” Lateral excitatory, inhibitory, and afferent weight connections are normalized separately.

In the neural network theory, the connection weight between two neurons is considered as a parameter that can be adjusted to optimize the performance of the network. This process of parameter adaptation is called learning. In biological terms, it may refer to synaptic changes during development (Gerstner and Kistler, 2002). The famous Hebb postulate (Morris, 1999) is phrased as synaptic changes are driven by the correlated activity of pre- and post-synaptic neurons. Experimental evidence (Tsien, 2000) suggest that the correlation-based synaptic adaptation processes are involved in neural plasticity. The mathematical formulation of Hebb's rule also called correlation-based learning is an interest of our study because of three aspects: locality, cooperativity, and competition. Locality means a change in the synaptic connection depends on local variables. Cooperativity implies that the pre and postsynaptic neurons have to be active simultaneously for synaptic weight change to occur. Competition is essential for any form of self-organization and topographic pattern formation, where weights of a certain subgroup of synapses are strengthened at the expense of others. In simulations, competition can be implemented by inhibitory interactions and the normalizing sum of all weights converging onto the same postsynaptic neuron (Gerstner and Kistler, 2002). Hebb's original postulate does not contain a rule for a decrease of synaptic weights. In such a system all weights saturate at maximum value. To make learning rule more competitive and useful divisive normalization was proposed (Miikkulainen et al., 2006) where each weight is intended to scale down in proportion to its original value. They also stated that initially normalization terms were introduced for a computational reason (Rochester et al., 1956) but many works (Turrigiano, 1999) has uncovered a number of neural regulatory mechanisms within the cell that regulate the overall synaptic strength during adaptation. There are many variants of Hebbian learning rule (Gerstner and Kistler, 2002). STDP is one variant of Hebbian learning where synaptic weight gets strengthen if presynaptic neuron fires just before postsynaptic neuron. Another variant is an asymmetric Hebbian rule (Schulz and Reggia, 2004) and closely resemble the experimentally observed temporal asymmetry embodied in the Spike-Time-Dependent Plasticity (STDP) (Fu, 2004; Caporale and Dan, 2008).

#### General Procedure Used to Model the Parameters

All the parameters were chosen through systematic manual trial and error exploration (Table 1). For each parameter set, a model with initial random connections was trained and check for the unique spatial representation for each of the input sequences. The parameters that transform different input sequences into very similar spatial representations are discarded.

While conducting a simulations **r**_exc_, **γ**_inhb_ are fixed at 3 and 1 and varied **r**_inhb_, **γ**_exc_ systematically to find the suitable parameter values. A parameter is said to be suitable if the model learns to spatially represent the sequences in the train set uniquely. **r**_inhb_ is set to global (the maximum allowable radius in NF) initially and reduced in steps of 2. Initially, **γ**_exc_ is given such a value that assures excitatory-inhibitory balance. When building a computational model, assumptions must be made about biological processes that are not well-understood. The above assumption was also made out of computational necessity and has not been characterized experimentally. The afferent connection strength **γ**_aff_ is set to 1, except in the third simulation. Here **γ**_aff_ is set to 0.3 to reduce the effect of fixed afferent connection on initial activity. All the three learning parameters (**α**_aff_, **α**_exc_, **α**_inhb_) take the same value and are chosen as 0.05. Each moving stimulus is created with a set of images/frames of size 64 × 64. **RF** is chosen randomly based on the simulation. Using the parameters **Image size** and **RF**, NF dimension was calculated as:

(6) $$\begin{array}{r} Dim=\;\frac{Image\;size-RF}{stride}+1 \end{array}$$

where stride = Number of pixels through which we slide the filter at every step

The systematic exploration of varying parameters one at a time showed that the parameters such as α_aff_, α_exc_, α_inhb_, and settling time are less sensitive and result in a network that is robust to small changes. However, The parameters r_exc_, r_inhb_, γ_exc_, γ_inhb_ that controls the influence of excitatory and inhibitory inputs, are relatively sensitive and need to fit in the given temporal sequence.

#### Generating the Topographic Map of Neuron Responses

Neurons in the trained network respond selectively to the direction of motion feature. The preferences of each neuron often vary systematically across the sheet of neurons in the NF revealing an underlying topographic structure. Also, due to the push-pull effect of lateral interactions, short-range excitation ensures correlated activity to similar stimuli over nearby neurons and anti-correlated response over long distances. This effect assembles the neurons within the NF into small patches and each patch becomes active in the specific direction of stimulus motion. Such cortical maps were delineated experimentally in monkeys striate cortex (Blasdel, 1992).

The set of all time-varying stimuli was presented to the trained network to determine the neurons' preferred direction of motion. A neuron is said to be preferred to the specific direction of the motion of the stimulus if and only if the stimulus is effective in achieving a maximum response in the neuron. Each neuron's preferred direction of motion was used as an entry in the map.

## Results

### Single NF Simulated Using Moving Bar Stimuli Shows Direction Selective Responses Analogous to Those of V1 Cells

**Figure 2:**
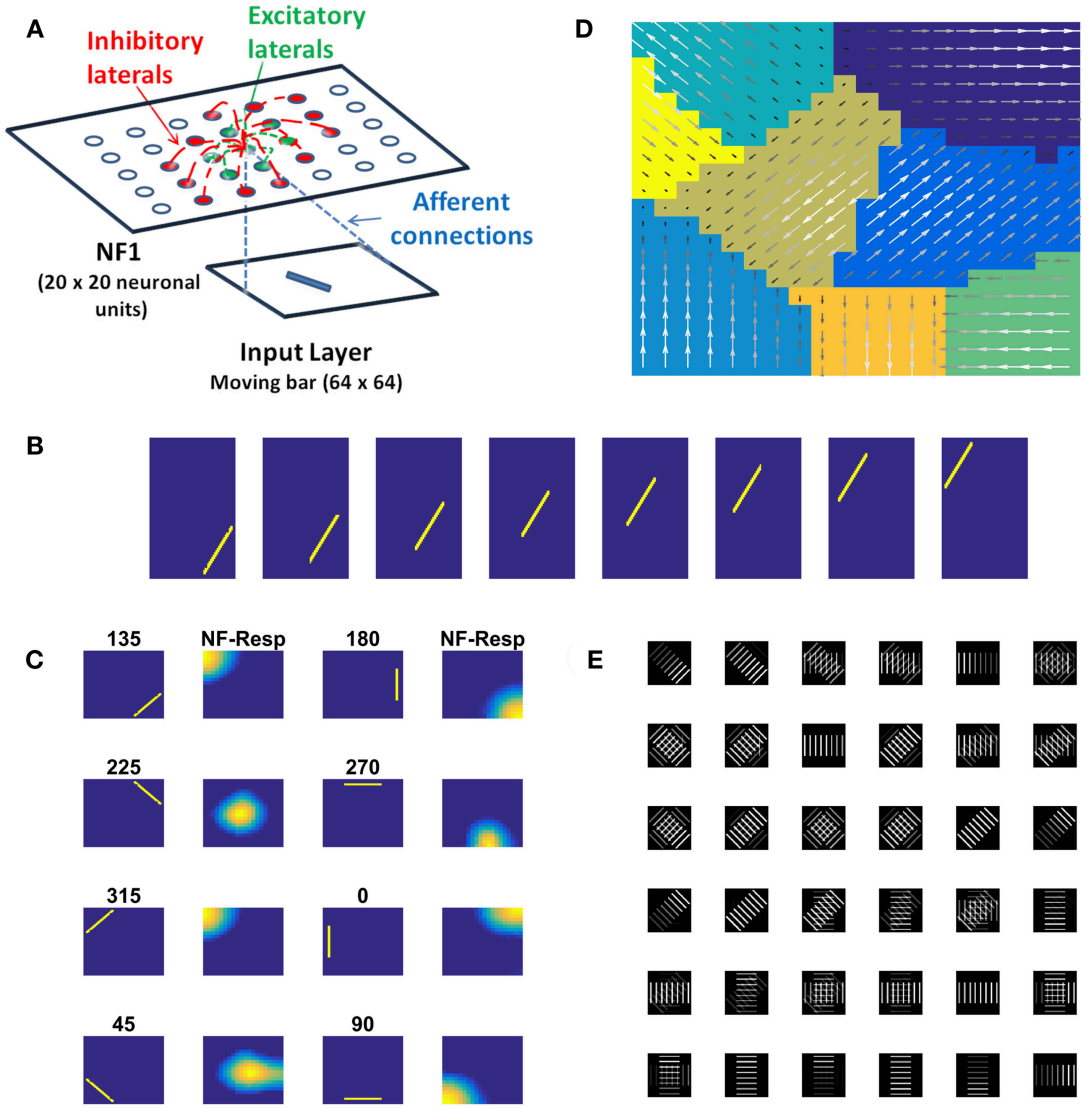
Direction sensitivity. **(A)** *The Architecture used to simulate the direction sensitivity of V1 cells*: The model consists of two stages: (a) an input layer where moving bar is presented (b) NF (20 × 20 units) analogous to V1. Green arcs represent the excitatory connections and the red arcs represent the inhibitory connections. The afferent connections are represented with blue dotted lines **(B)**. *Sample bar stimulus moving in 135*°: the bar of size 30 × 2 pixels are placed on 64 × 64 pixels black background and is made to move in 8 directions with the direction of motion perpendicular to the orientation. The motion is captured in a sequence of 8 frames. **(C)** *Network response to moving bar stimulus after 500 epochs of training*: the first and the third columns display the first frame of the moving bar sequence, the label above it shows the direction of motion of the bar. The response of NF has plotted in the second and fourth columns. Each input is mapped to the unique spatial position on NF. **(D)** *Direction selectivity map*: Direction selectivity map is plotted using the convention described in the section “Generating topographic map.” We observed that the patch of neurons selective to one direction of motion often has an adjacent patch with opposite direction preference. The arrows indicate the direction preferences developed by the neurons on NF. The arrow with the highest magnitude indicates the peak response of the neuron **(E)**. *The afferent weights developed by the selected neurons in NF*: Initial afferent weights are random. After training Gabor like afferent weights are developed. Different varieties of tuned afferent weights (64 × 64 pixels each) are selected from the whole population (Figure S1) and displayed here.

In this study, we construct a direction sensitivity map by training a single NF, using a set of sequences of a moving bar pattern. The architecture of the network used for this purpose is shown in Figure 2A, where input images are presented in the input layer, which is then used to stimulate responses in the NF. NF size, number of epochs and other network parameters used in the simulation are shown in Table 1.

The training set consists of 8 sequences of a bar moving in 8 directions: 0, 45, 90, 135, 180, 225, 270, and 315°. For instance, in 0°, the bar is placed in vertical position and is moved from left to right. Complete details about the stimuli generation are given in the Methods section.

During the training, each moving bar sequence (Figure 2B) was drawn randomly and presented to the network frame after the frame. Training was carried out as described earlier. Next we examined the response properties of the neurons by plotting the network activity (Figure 2C) to the bar sequence moved in 8 directions: 0, 45, 90, 135, 180, 225, 270, and 315° The activity patch under “NF-Resp” column denotes the population of neurons fired to a given drifting bar. Eight different population bubbles were seen, each specifying its preference to a specific direction of motion. Some populations were overlapped (for example 135 and 315°, 225 and 45°), indicating that some neurons have a preference for more than one direction of motion. Such multiple preferences can be seen in the case of stimuli having different directions of motion with the same orientation.

Direction selectivity map with the neuron's best preferences is plotted in Figure 2D. The color patches indicate a different population of neurons has different direction preferences. The arrows indicate the neuron preferred directions and the magnitude indicate the neuron activity. Almost all adjacent color patches have opposite direction preferences. For instance neuron patches preferential to 135 and 315° are adjacent. Similarly, patches preferential to 45 and 225° are adjacent.

Figure 2E shows the developed afferent weights for the selective neurons. Initial afferent weight values were random and were bounded between 0 and 1. During training, these random weights were self-organized in such a way that the neurons that have the same orientation and opposite direction preferences were pruned as a continuous patch and seen as four big patches in response to 8 moving stimuli. In each patch, neurons were clustered into two subgroups with opposite direction preferences. As shown in Figure 2E some neurons afferent weights are tuned to the specific direction of bar motion, others, particularly neurons present at the boundaries of the patches, showed tuned weight preferences to more than one motion direction. These results were inconsistent with experimental studies (explained in the Discussion section).

### Component and Pattern Motion

**Figure 3:**
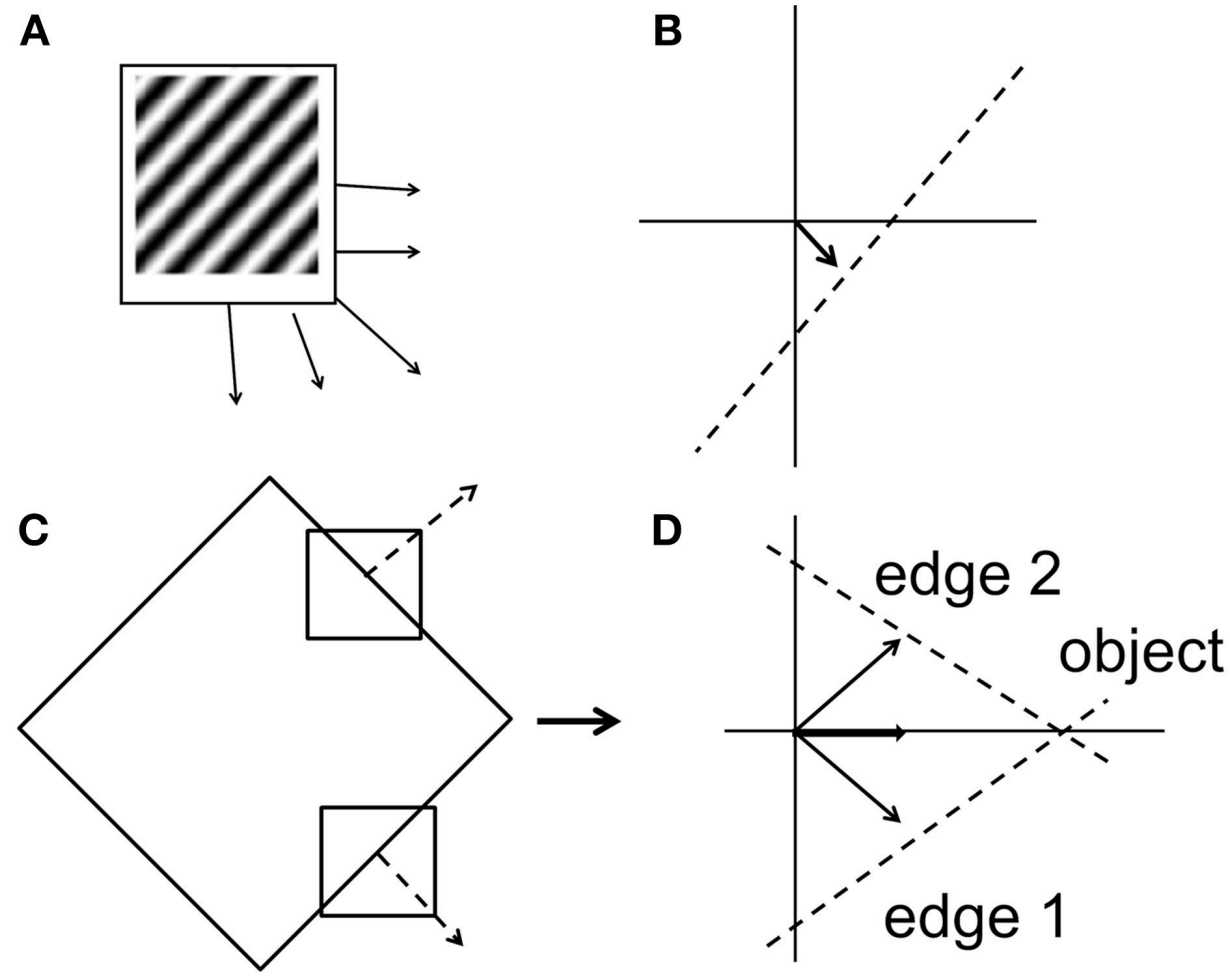
Aperture Problem. **(A)** A grating pattern consisting of alternating black and white bars. The grating is allowed to move in different directions. Thin arrows in **(A)** represent the set of physical motions of the grating pattern in various directions. The motion of all these grating patterns is indifferent when viewed through a small window, and this motion direction is perpendicular to the orientation of the grating (as a thick arrow shown in **B**). This ambiguity in determining the direction of motion of the grating is termed as aperture problem. In case of motion of a two-dimensional object (e.g., square or diamond), local motion cues (dotted arrows show in **C**) are divergent and are very different from the actual object motion. In **(D)** thin arrows represent the local motion of each edge seen through RF. An intersection of two constraint lines from both the edges represents the true motion of an object (thick arrow in **D**).

In case of a moving 2D object, parts of its boundary seen through narrow apertures seem to move in various directions, quite different from the direction of motion of the entire object. This problem is referred to as the aperture problem (Figure 3; Fennema and Thompson, 1979; Wuerger et al., 1996; Pack et al., 2001, 2003). The motion of the boundary segments is called *component motion* while that of the whole object is called *pattern motion*. Electrophysiological studies suggest that while V1 neurons respond to the component motion, neurons of MT respond to pattern motion (Rodman and Albright, 1989; Priebe et al., 2003; Bradley and Goyal, 2008). The problem of computing pattern motion from local component cues has been studied extensively using computational modeling (Rust et al., 2006), Psychophysics (Adelson and Movshon, 1982; Movshon and Newsome, 1996), functional Magnetic resonance imaging (Huk and Heeger, 2002), and single unit Electrophysiology (Movshon and Newsome, 1996).

### Two-NF Network Simulated Using Moving Two-Dimensional Object (Plaids, Solid Square) Sequences Show Pattern Selective Responses

We now propose an expanded version of the direction sensitive architecture to model component and pattern selectivity. The proposed hierarchical pattern selectivity model has 3 stages: input layer followed by two NFs (as shown in Figure 1A), corresponding to V1 and MT. We simulated the network with two types of input stimuli: (i) moving the solid square, and (ii) moving plaids, and showed that the neurons in NF1 respond to the direction of component motion (edges, gratings) while those in NF2 respond to the direction of pattern motion (square, plaids).

The training set consists of 2D patterns (square, plaid) moving in 8 directions: 0, 45, 90, 135, 180, 225, 270, and 315°. Complete details about stimuli generation and the parameters used in the simulations are given in the Methods section and Table 1 respectively.

#### Case 1: Moving Solid Square

**Figure 4:**
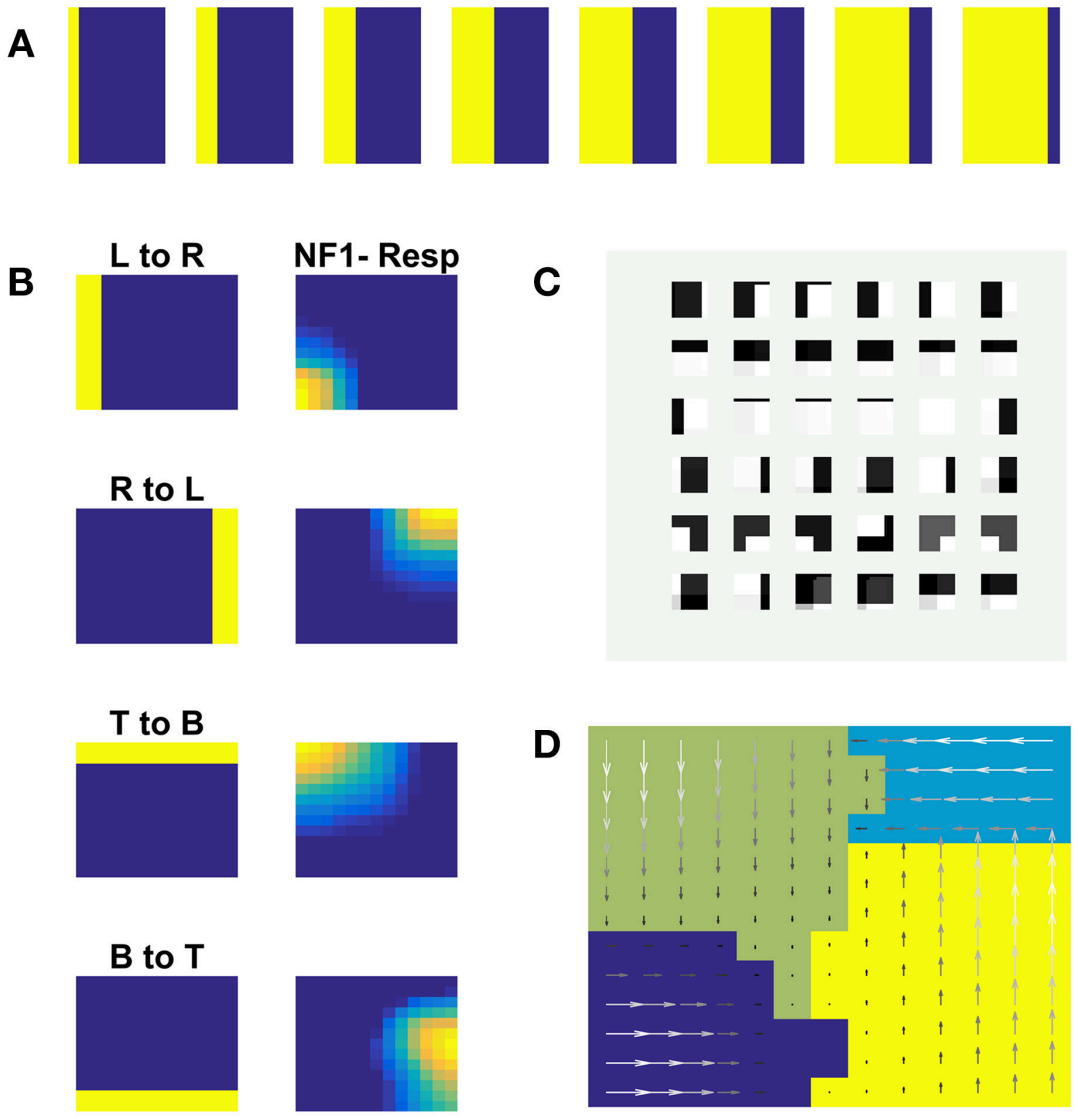
NF1 neuron preferences to moving edge stimuli. NF1 of the two-NFs network is trained using moving square stimuli. 24 × 24 pixel white square is moved on 64 × 64 pixel black background. As neurons in NF1 has small receptive fields (12 × 12 pixels), at any instance, it can see a part of a square and become selective to local motion cues also called component motion which is an edge motion in this case. **(A)** Sample input of an edge (64 × 64 pixels) moving from left to right. An edge can be moved in four possible directions [left to right (L to R), right to left (R to L), top to bottom (T to B) and bottom to top (B to T)] and the response of NF1 to an edge motion is displayed in **(B)**. Even though NF1 is trained using moving square objects, most of the NF1 neurons tuned to local edge motion (i.e., component motion). **(C)** Depicts the trained afferent weights (12 × 12 pixel each) for the selected neurons. **(D)** Topographic map formed out of NF1 response to edge motion: The arrows indicate the neuron preferences in the direction of edge motion.

NF1(13 × 13 units) was trained using moving square stimuli whose frame size is 64 × 64 pixels and square size is 24 × 24 pixels. The RF of NF1 neuron is of size 12 × 12 pixel. Hence at every instance, NF1 neurons either look at part of a square or no square at all. The parts of a square are horizontal and vertical edges which are also called its components. Due to the smaller receptive fields, NF1 neurons encode only that local motion direction that is orthogonal to edge orientation. As result, NF1 neurons become selective to 4 directions of an edge motion (0, 90, 180, 270°) even though the square moved in 8 directions. To verify that the NF1 neurons respond to the component motion in the input sequence, we created moving edge stimuli that move in four directions (left to right, right to left, top to bottom, and bottom to top). Each moving edge stimulus is made up of 64 frames with frame size 64 × 64 pixels (i.e., for each time step the edge moves one pixel ahead). Eight sample frames of edge moving from left to right are shown in Figure 4A. The responses of NF1 neurons (that was earlier trained using moving square stimuli), to the 4 moving edge stimuli are displayed in Figure 4B. The figure shows four independent neuronal populations, each is selective to the specific edge motion. Figure 4D depicts the direction selectivity map to the edge moving in four directions. Figure 4C represents tuned afferent weights of NF1 selected neurons. We observed that the afferent weights of NF1 neurons were tuned to the direction of motion of an edge.

Next, we train NF2 keeping NF1 weights fixed. The moving square stimulus was presented to the network frame by frame. The NF1 neuron responses (the local component cues) were presented as input to NF2 neurons. Training was carried out for 500 epochs. We observed that the NF2 neurons are selective to a specific direction of square motion.

**Figure 5:**
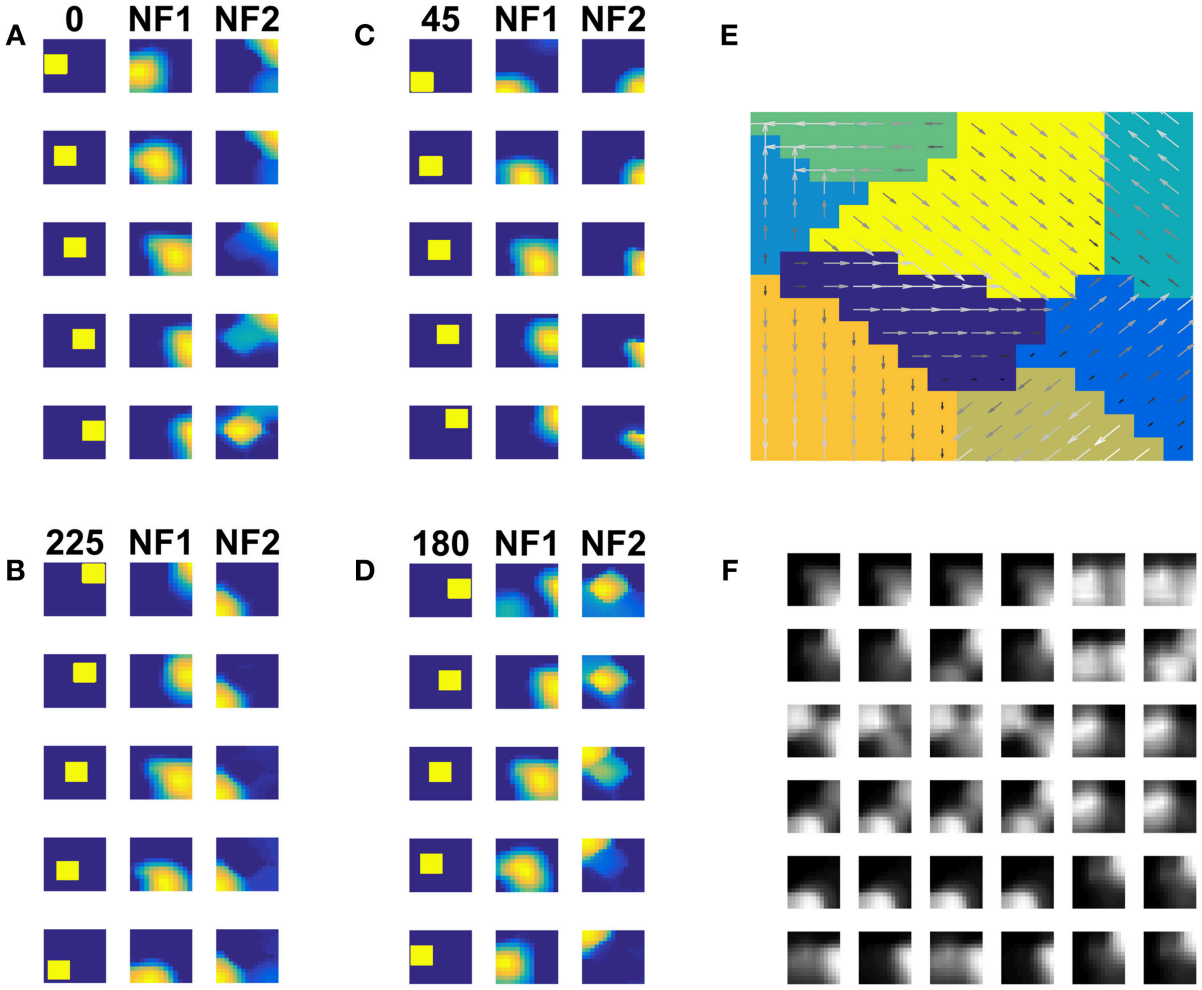
NF2 response to moving Square stimuli. **(A–D)** are four clusters. In each cluster first column depicts the frames of moving squares stimuli (64 × 64 pixels), and the corresponding activity on NF1 (13 × 13 units), and NF2 (15 × 15 units) are shown in the next two columns. The label on the first column represents the direction of motion of a square object (A: 180°, B: 45°, C: 0°, and D: 225°). Neurons in NF1 respond to local motion cues. At each frame presentation, different neurons receive afferent input from the square object and become active, according to its preferred direction of motion, thus the activity pattern follows the square stimulus. In NF2 neurons are selective to the entire object motion (also called pattern motion) by aggregating local motion cues from NF1. Nearly stabilized activity can be seen over the presentation of the whole moving square sequence. Different patches of neurons uniquely become selective to different directions of square motion. **(E)** Shows the pattern selectivity map plotted out of NF2 neuron responses to moving square stimuli. The arrows indicate the neuron preferences to 8 motion directions: 0, 45, 90, 135, 180, 225, 270, and 315°. The magnitude of the arrow represents the activity of the neuron. Peak activity is represented by neurons with the highest magnitude. **(F)** Represents the NF2 afferent weights (13 × 13 pixels each) of the selected neurons. It shows that the NF2 neurons developed spatiotemporal receptive fields in the direction of pattern motion.

We inspected the development of pattern selective properties of the NF2 neurons by computing the network response to a two-dimensional moving object (square). Figures 5A–D displays the network responses to four moving square stimuli. Each cluster depicts the firing patterns of neurons in NF1 and NF2, in response to the presentation of a moving square sequence. The square pattern was translated spatially from one end to another across the frames. Accordingly, NF1 firing pattern (as shown under NF1 column in Figures 5A–D) also displaces, since the neurons here encode the edge motion seen within the RF. In NF2 (as shown under the NF2 column in Figures 5A–D), the activity pattern is stabilized across the frames and the corresponding neuron population is found to be encoded uniquely the true direction of stimulus motion. The pattern selective properties of NF2 neurons are abstracted as a map in Figure 5E. Like neurons in the direction selectivity map of Figure 2D, here also NF2 neurons preserve topography. That is, the patch of neurons responding to a certain direction of motion often have adjacent neuron patch with firing preferences to the opposite direction. Trained afferent weights for the sample of NF2 neurons are plotted in Figure 5F.

#### Case 2: Moving Plaids

**Figure 6:**
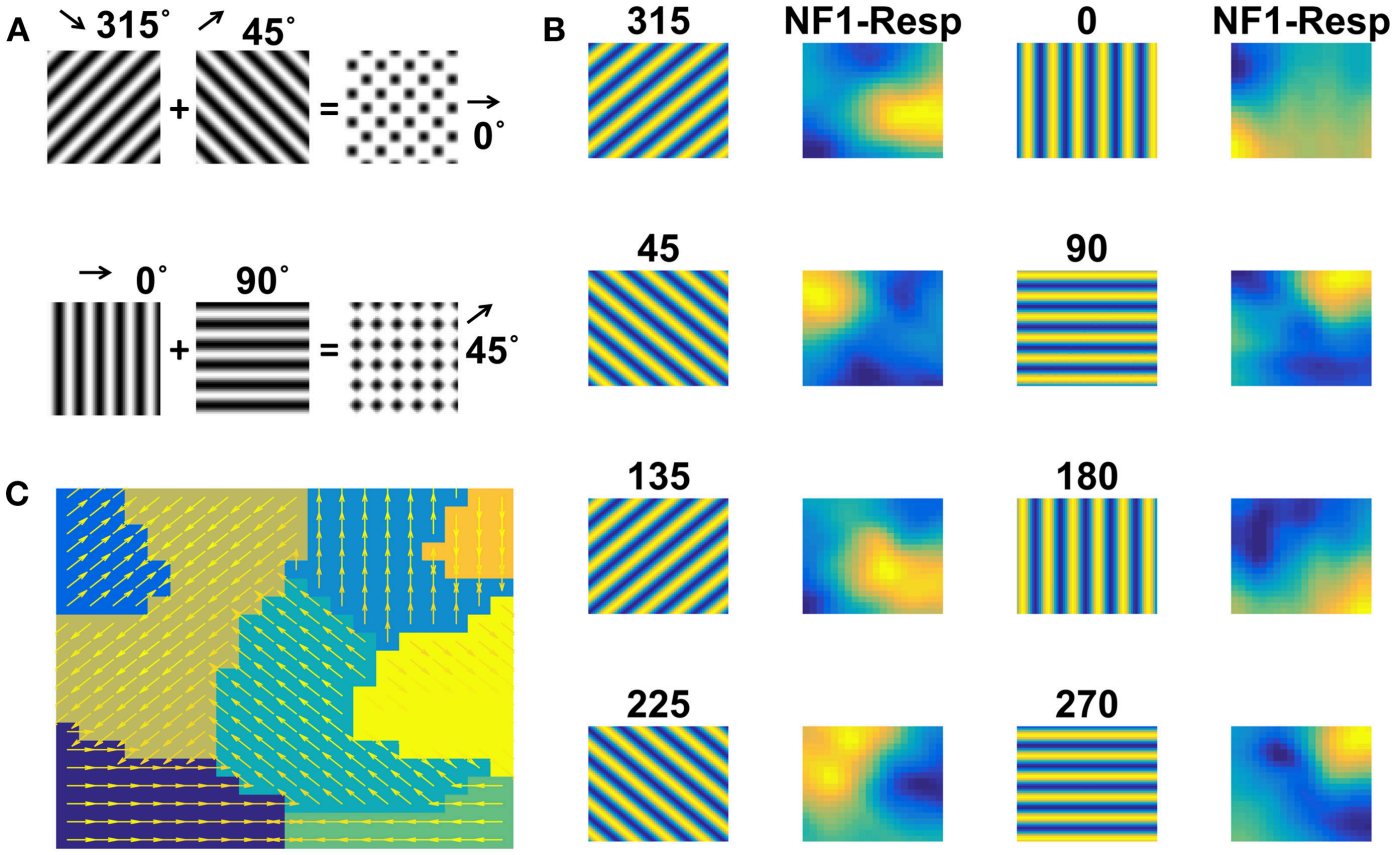
NF1 response to moving grating stimuli. Grating stimulus consists of alternating black and white bars. Gratings (64 × 64 pixels) are moved in 8 different directions such that the direction of motion is orthogonal to the grating orientation. Plaid stimulus moving in 0° is created by superimposing two gratings moving in 45° and 135° as shown in **(A)**. NF1 (20 × 20 units) is trained with moving grating stimuli for 1,500 epochs and the response is plotted as shown in **(B)**. Here the first and third columns display the frames of moving grating. The label above it indicates the direction of motion of the grating. The second and the fourth columns represent the neuronal preferences to a given grating. As seen in other simulations, different neuron patches become active to different motion directions. Also, component selectivity map is shown in **(C)**. The arrows indicate the neuron preferred directions of motion.

Moving gratings and moving plaids are created as described in the Methods section. NF1 was trained with sinusoidal gratings moving in 8 directions. The trained network response is shown in Figure 6B. Eight different firing responses are shown, each corresponding to a specific direction of motion grating. Also, overlapped populations are noticed in case of drifting stimuli with similar orientations and opposite motion directions. The component selectivity map to moving gratings is depicted in Figure 6C.

**Figure 7:**
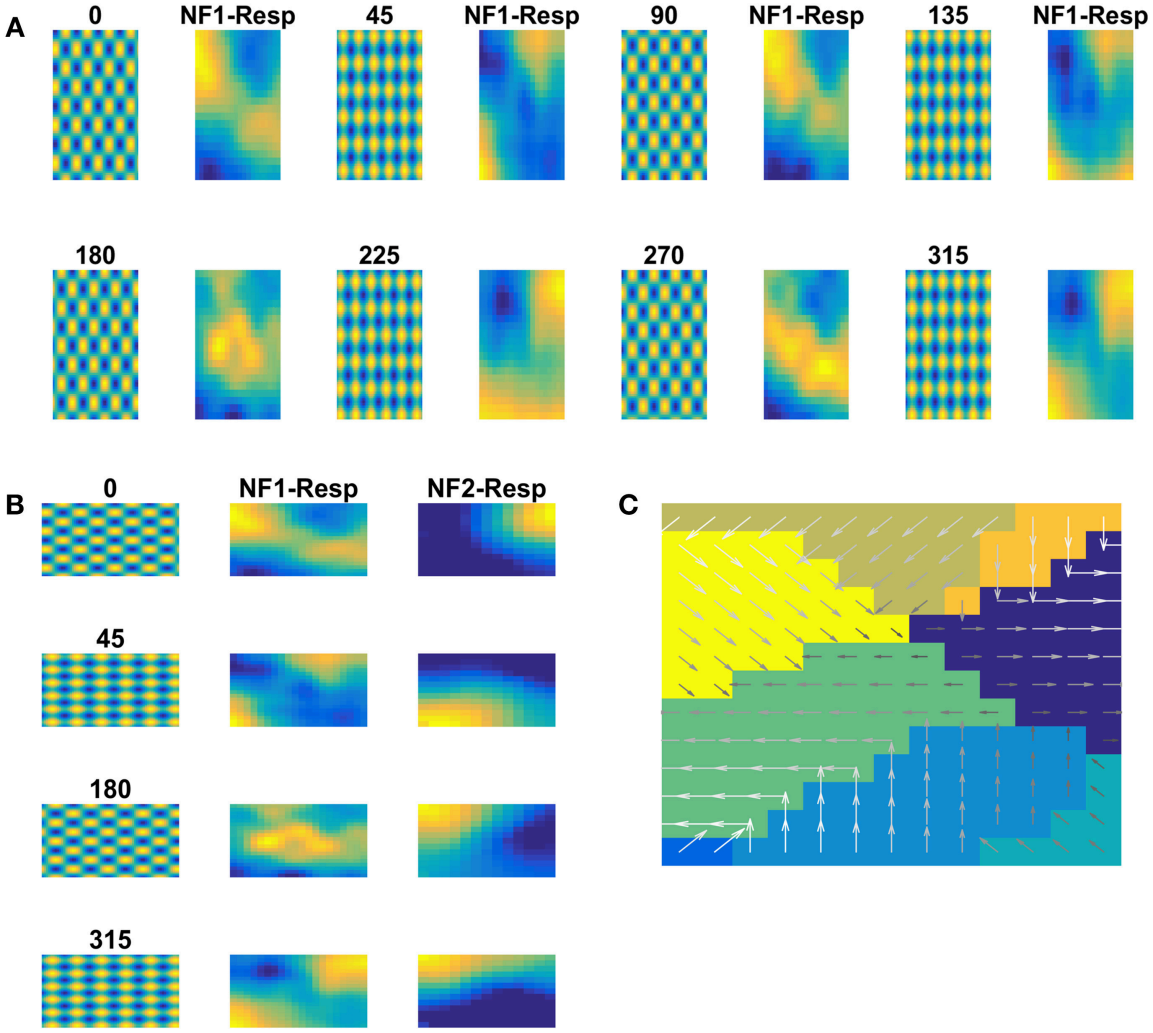
Two-NFs network response to moving plaid stimuli. the Plaid (64 × 64 pixels) stimulus is created from its components (two gratings) and is allowed to move in 8 different directions. NF1 (analogous to V1) is trained with plaid components (i.e., moving gratings) and its response to moving plaid stimuli is plotted in **(A)**. First, third, fifth, and seventh columns display a frame in moving plaid sequence. The label above it indicates the direction of motion of a plaid. Second, fourth, sixth, and eighth columns represent the NF1 response to plaids, and two neuron populations are active in response to every moving plaid stimulus. As each plaid is composed of two gratings, neurons that are preferential to these moving gratings are becoming active. For example, the plaid moving in 315° is made from gratings moving in 270 and 0°. The activity pattern of these two plaid components (shown in Figure 6B) gets integrated and produces a plaid response as two activity bubbles. NF2 (analogous to MT) is trained using plaid pattern moving in 8 directions, by keeping NF1 weights constant. The response of NF2 to four sample stimuli is shown in **(B)**. The first column represents frames of moving plaid stimuli, second and third columns labeled as NF1-Resp (20 × 20 units) and NF2-Resp (13 × 13 units) represents the responses of NF1 and NF2, respectively. We observed that in response to 8 moving plaid stimuli 8 different patches of neurons become selective to different directions of motion, and the corresponding pattern selectivity map is shown in **(C)**.

Now the question is: Does NF1, trained using moving grating stimuli, respond to the direction of plaid components by extracting them from the moving plaid stimulus? To this end, we examined NF1 responses to moving plaid stimuli, which is constructed by superimposing two orthogonal moving gratings (chosen from the training set used to train NF1) separated by 90° (Figure 6A). As shown in Figure 7A (under column NF1-Resp) two distinct activity bubbles are observed in response to the moving plaid stimuli. To verify whether these response profiles derived exactly from the same two gratings used to construct the plaid, we compared it to Figure 6B. We were able to ascertain that the NF1 neurons that were trained using moving grating stimuli will produce two distinct population responses; each is corresponding to the moving gratings using which the moving plaid was made of.

We proceed to train NF2 using moving plaid stimuli, with NF1 weights kept constant. We illustrate the response properties of trained NF2 neurons in Figure 7B. We observed that distinct widely separated clusters of neurons become selective to each moving plaid stimulus. The neuron preferences to different directions of moving plaids are displayed as the pattern selectivity map (Figure 7C).

### Three-Layer Network (With Two NFs) Simulated Using Translated Random Dot Stimuli Shows Translational Flow Selective Responses

**Figure 8:**
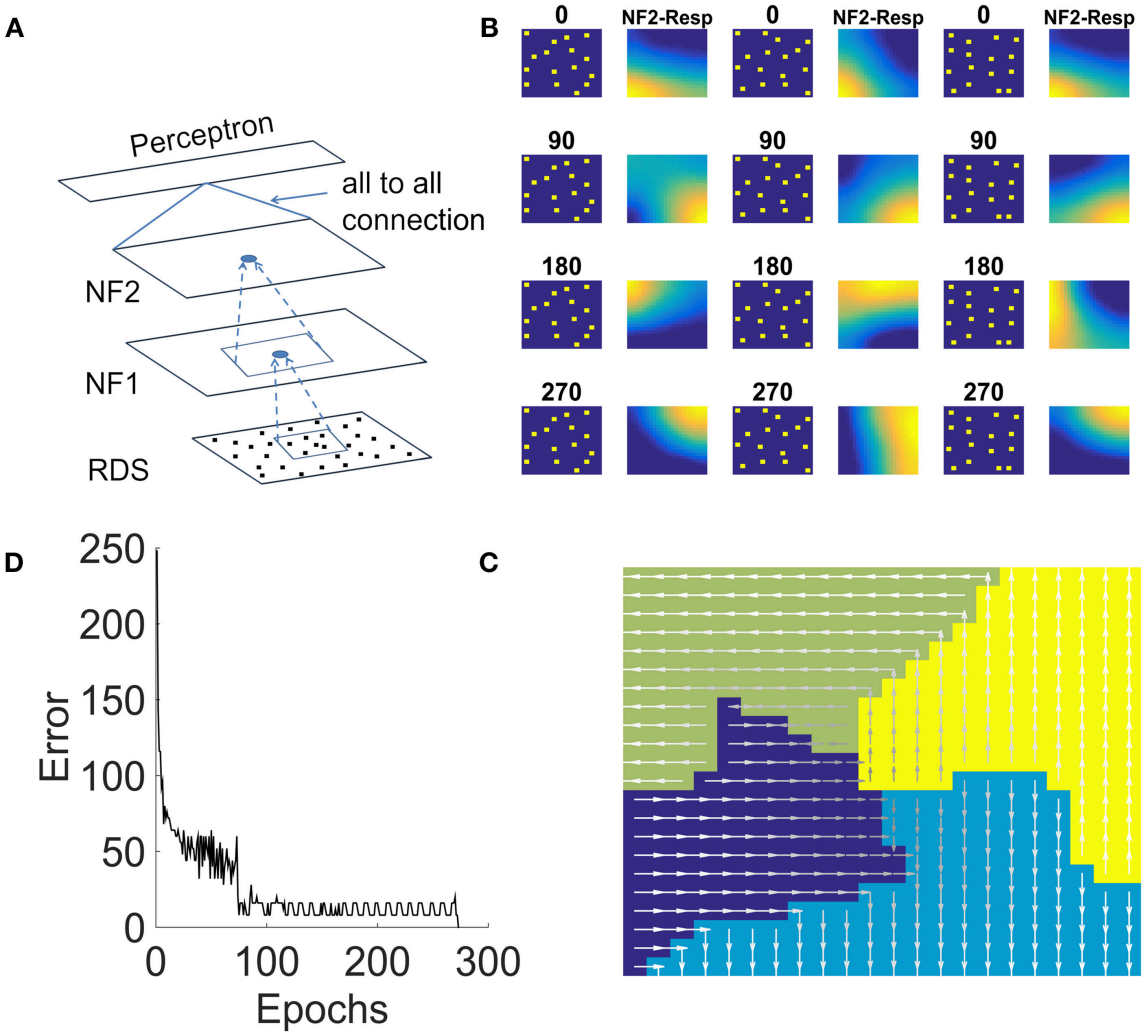
NF2 response to translational random dot stimuli. **(A)** Proposed 2NFs *network*: both NFs are trained using unsupervised asymmetric Hebbian rule and the third single layer perceptron is trained using backpropagation. Random dots stimulus (RDS) is created by placing tiny squares of size 2 × 2 pixel (assumed as dots) on 32 × 32 pixel size grid randomly with a constraint that each 8 × 8 pixel grid can accommodate only one dot. Thus, 16 dots are placed randomly and moved dots coherently in 4 directions: 0, 90, 180, 270° to create translational flow sequences. Thus, each dot configuration creates 4 sequences for the train set. First, third and fifth columns in **(B)** shows three different dot configurations moving in the same direction. Second, fourth and sixth columns show the NF2 activity, when these configurations moved in 4 directions. Here the neurons encode the coherent motion direction, independent of the precise dot configuration. **(C)** It represents the translational flow selectivity map in response to the train set consisting of 80 sequences. The arrow direction indicates the neurons preferred direction of motion to the translational flow stimuli. **(D)** Error plot obtained while training single layer perceptron using NF2 responses of the train set. Single layer perceptron has an input layer and an output layer; the weights (all-to-all connections) between them are trained using regular backpropagation. Perceptron took nearly 300 epochs to learn the input.

In this study, we present an extension of the model of the previous study to respond to translated random dot patterns. The architecture of the network used for this purpose (shown in Figure 8A) is similar to the earlier study except that it consists of a single layer perceptron above NF2, which receives input from NF2 in fully connected fashion and was trained using backpropagation. Network properties and the parameters for NF1 and NF2 are fine-tuned according to the present study. More details about the size of the NFs, the number of epochs and other scaling and learning parameters used in the simulation are shown in Table 1.

The stimulus of this study, a translational flow sequence, was created by moving randomly placed tiny squares (assumed as dots) coherently in 4 directions: 0, 90, 180, and 270°. Sixteen tiny squares, each of size 2 × 2 were placed randomly on a 32 × 32 matrix. We assumed it as dot configuration. Twenty five such random dot configurations were created and each of those configurations is translated in four directions to create 100 translational flow sequences. Out of these, 80 sequences were used for training and the remaining 20 for testing. Complete details about flow stimuli generation were furnished in the Methods section.

During training, each translational flow sequence from the training set was drawn randomly and presented to the network frame after frame. The two NFs in the network were trained one by one as is described in the previous sections. A lower NF was first trained to saturation before the next NF is trained. We fixed afferent weights of NF1 as “1” and maintained them as constant throughout the simulation. This small variation was adapted to ensure the NF1 neurons encode position independent motion selective responses. NF2 afferent weights are random initially and were adapted during training.

We examined the response properties of the trained neurons in both the NFs by plotting the network response to the training set. Figure 8B shows the response of the NF2 neurons to the selected configurations of the training set. It can be observed that in NF2 four different neuron clusters were formed each is selective to the specific direction of translational flow and is independent of dot configuration. The resulting NF2 response of the 80 sequence training set is abstracted as a translational flow selectivity map as shown in Figure 8C. The arrows indicate the preferred direction motion of the neurons.

Generalization capability of NF2 neurons was verified by presenting a test set to the network. We observed that the activity pattern appeared in both the NFs is nearly similar to the activity pattern seen for the training set. To quantify these observations, we added a single layer perceptron network (acts as a classifier) as an additional layer above NF2 and are trained using NF2 neuron responses of the training set. Training was carried out for 300 epochs and the corresponding error bar is shown in Figure 8D. The trained perceptron network successfully classified translational flow sequences into 4 directions with an accuracy of 100 % on the training set and 90% on the test set with 2 misclassifications.

### Model Behavior in Response to Variations in r_exc_, r_inhb_

Neurons in the neural field (NF) receive initial activity as a weighted sum of input. Each input causes initial activity in many neurons, and most of this activity is redundant. To achieve efficient coding this redundant activity must be reduced where the role of lateral interactions come into the picture. Lateral inhibition introduces competition among the neurons by de-correlating activity between distant neurons in the NF and increasing correlation among nearby neurons. In the simulations, these effects were controlled by 4 parameters: **r**_exc_, **r**_inhb_, **γ**_exc_, **γ**_inhb_.

Case 1: If **r**_exc_ is too small (e.g., <3) small neuron populations respond to each stimulus. This result in the inefficient use of available map space and smooth topographic maps cannot be produced.
Case 2: If **r**_inhb_ is low (e.g., close to **r**_exc)_, decorrelation between distant neurons decreases and the correlation between nearby neurons increases (due to high excitatory), results in highly saturated response spreads across the sheet. Most of the neurons have preferences in multiple directions. Thus, during training inputs are transformed into overlapped spatial representations.
Case 3: If **r**_exc_ is too high (e.g., half of the network space), a large population of neurons responds to each stimulus, resulting in redundant coding. Different input sequences transform to same spatial representations
Case 4: Too high **r**_inhb_ (global inhibition) results in the elimination of excitatory activity during settling. As a result, none of the weights get updated in response to the input sequence. Training will not take place.

The same effects can be achieved in small scales by adjusting overall strength of excitatory and inhibitory effects represented by **γ**_exc_, **γ**_inhb._ In most of the simulations, **γ**_inhb_ is set to 1 and the only **γ**_exc_ is varied.

### Decoding Stimulus Information From the Neuronal Responses of the Trained Network

In all the simulations described above, we showed that the network response and its corresponding map can encode the direction of the moving stimuli. The proposed hierarchical feedforward neural field model acts like encoder where the pixel-based visual representation is transformed into high-level neural population activity patterns. In data analysis terms, the proposed model is creating a spatial map of spatiotemporal input patterns. To quantify the efficiency of this mapping, we used a simple single layer perceptron network as a decoder. Perceptron is a supervised learning algorithm to classify only linearly separable data points (Minsky and Papert, 1969). Here perceptron is not the part of dorsal motion detection stream which we are modeling; rather it is a proof of principle to show that the inputs can be decoded from the abstract maps of the NFs using a linear classifier like perceptron.

**Figure 9:**
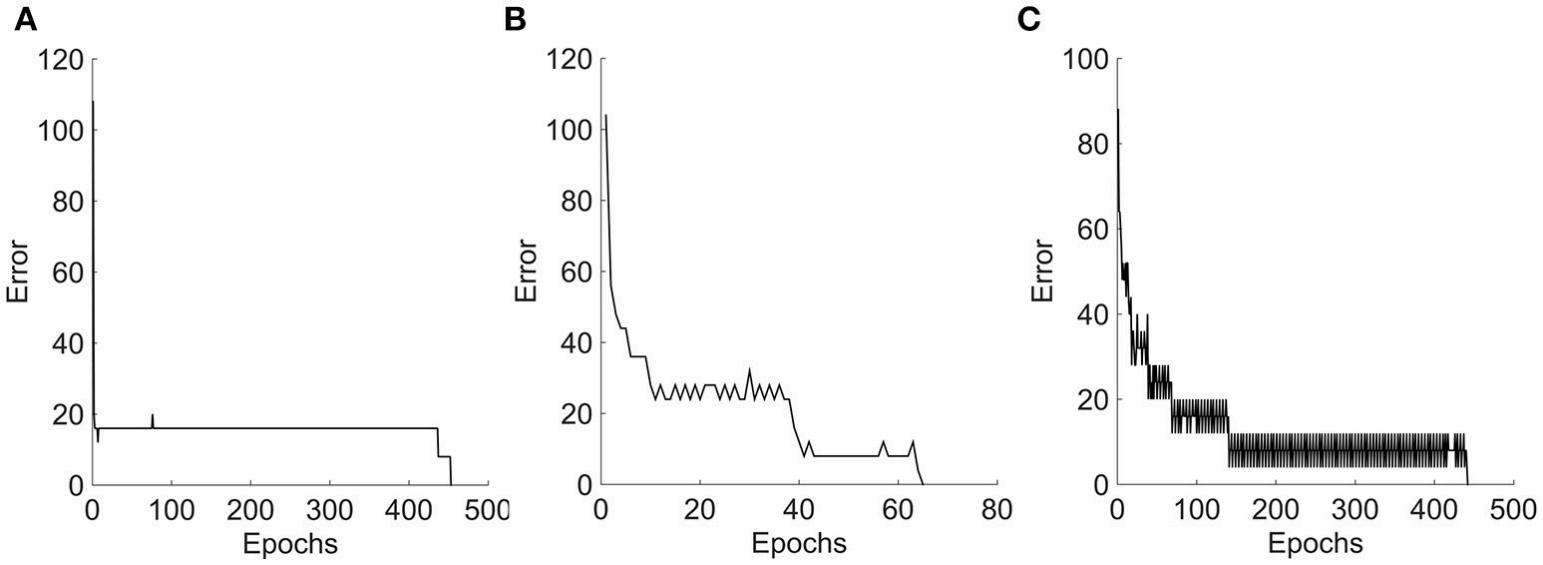
Error graphs obtained during the perceptron training. **(A–C)** Represents the error plots obtained for Bar, Square, and Plaids respectively, during the perceptron training. The NF layer encodes the motion information of moving stimuli as a unique neuronal population response over a network space. Perceptron takes this population values as input and learns the pattern in the input. The complexity of this response pattern is low to the bar and high to the plaids. The perceptron trained on less complex bar input converges with smooth error graph and the fluctuations were seen in the error graphs of the other two which were proportional to the complexity of the input.

Figure 9 represents the sum square error obtained during the perceptron training for the three tested stimuli. Three different learning curves represent the nature of information given to the perceptron network. In case 1: **moving bar** is a simple stimulus. This information is encoded by single layer neural field network, as a topographically ordered map. The perceptron learned this representation as shown in the error curve and converges at 500 epochs. In case 2: **moving square** is a two-dimensional object. A two-layer neural field network encoded it as a topographically ordered map, but it is less regular than that formed with bar. Fluctions seen in the error curve before the perceptron converges at 300 epochs, shows that the map generated is more complex than in the previous case. In case 3: **moving plaids** is more complicated input. A two-layer neural field network encodes this information in much more of complex map form. Perceptron trained with this input converged at nearly 500 epochs.

We made small modifications to the model from one simulation to other. With the simulation using moving square both NF1, NF2 are trained using moving square stimuli whereas in simulation using moving plaids, NF1 is trained using moving gratings and NF2 is trained using moving plaids. In the case of a square, NF1 encodes the direction of motion of an edge. As the square is moving on a black background, at any instance edge motion can be seen through the small receptive field that covers part of a square. NF1 need not be trained by creating a moving edge separately. However, NF1 that trained on plaids, cannot see the direction of motion of gratings from the plaid motion. Plaid moving in 0° was created by a pair of gratings moving in 45 and 315°. The NF1 trained using moving plaids can neither encode the direction of motion of gratings nor the direction of motion of plaids. Also with the simulation using random dots we, made variation to the afferent weights. All initial afferent weights are taken as 1 (unlike other simulations where they are random initially) and keep them constant throughout the simulation to make network learn only one feature, –that is the direction of motion, –and ignore the position information of dot. Due to such spatial homogeneity in the afferent weights, the neuron's response in NF1 is insensitive to the position of the dots.

### Robustness of the Model

In this section, we present the robustness of the trained network weights to various noisy stimuli and to the input of varying bar length. Two types of noises are added to the moving bar stimuli.

Salt and pepper noise is added to the training set with the initial noise pixel density 0.01. Fifty noisy sets were generated by increasing the noise pixel density up to 0.99 in steps of 0.02. The density 0.02 indicates 1% (40 pixels approximately) of the image pixels (64 × 64). To increase the noise density in the current noisy set, 1% of the non-noisy image pixels were made noisy by choosing them randomly. All these 50 noisy sets were presented to the network (trained earlier on non-noisy moving bar stimuli) in the sequence and the robustness of the trained weights are abstracted as a robustness index (RI) using the Equation (7). We observed that the RI value was decreased with the increase of noise pixels in the stimuli.

(7) $$\begin{array}{l} RI=\frac{1-\;Number\;of\;neurons\;deviated\;from\;its\;preferred\;direction\;of\;motion}{Total\;number\;of\;neurons\;on\;NF} \end{array}$$

Note that each neuron in the network that was trained earlier on non-noisy moving bar stimuli shows a high response to the specific direction of bar motion and this direction is considered as the preferred direction of that neuron.

Gaussian noise was added to moving bar stimuli with mean 0 and variance varied from 0.02 to 1 in steps of 0.02. Thus, 50 noisy sets were generated, presented to the trained network in the sequence and observed the decrease in the RI value with the increase of the noise variance.

**Figure 10:**
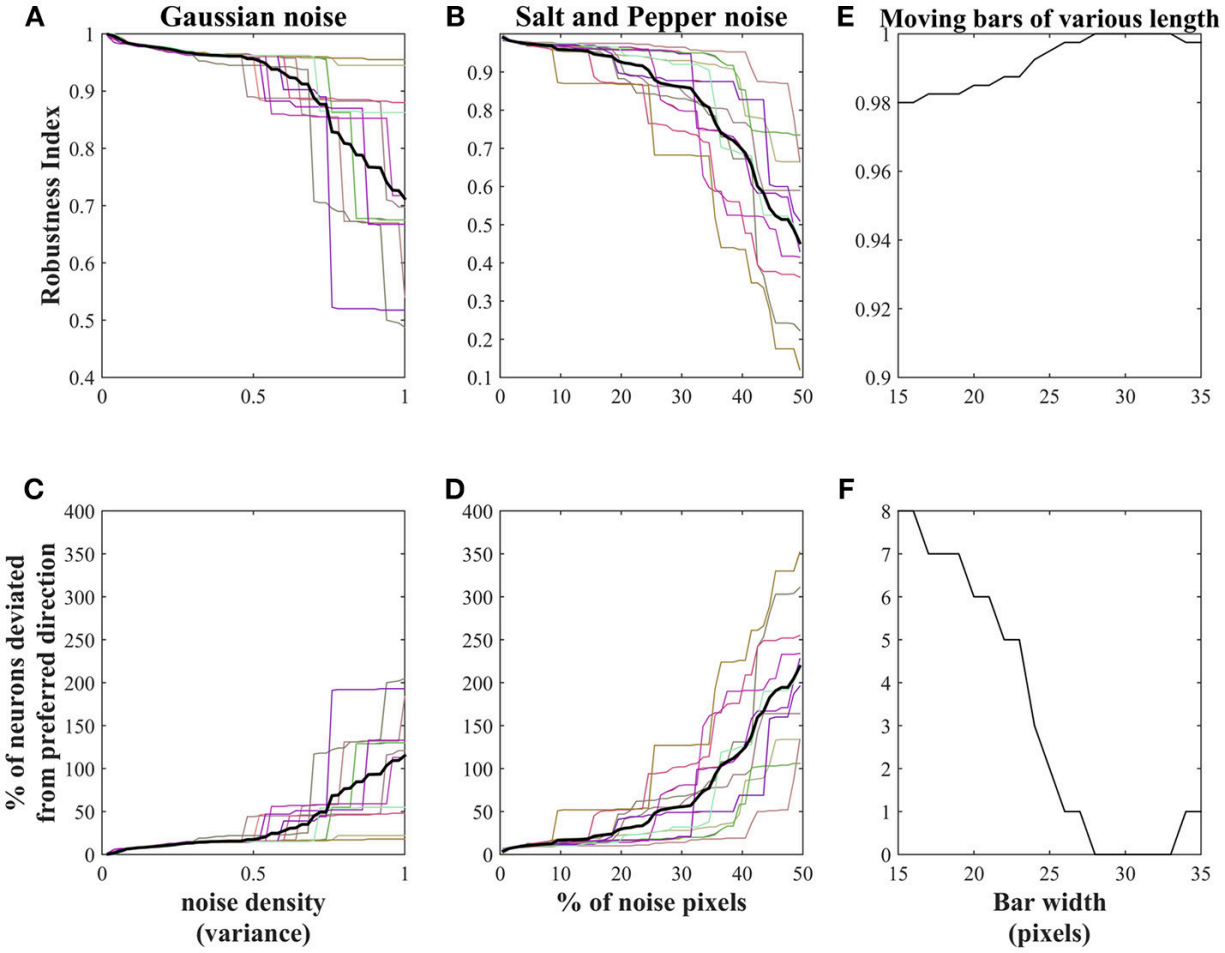
Robustness of the trained network: NF1(20 × 20 units) trained using non-noisy moving bar is used to test the robustness of the proposed network. **(A,B)** represents the decrease in the robustness index (RI)of the network with an increase in the noise density. The thick black lines in **(A,B)** indicates the RI average across 20 trials. In the case of salt and pepper noise, RI reaches zero when 50% of the training set pixels were made noisy. Similar results can be seen with Gaussian noise with variance = 1. The network shows high tolerance: to the Gaussian noise with a variance of <0.5 and to the salt and pepper noise whose density of <0.3. **(C,D)** represents the number of pixels deviated from its preferred direction in relation to the noise density. **(E)** represents the robustness of the network to the varying bar length. RI reduced slightly with a change in the bar length. **(F)** shows the number of neurons deviated from their preferred directions to the change in bar length.

The RI value calculated above indicates that the network is less tolerant of the highly noisy inputs. To know, the amount of noise allowed in the training set, to produce clear motion selective responses, we conducted 20 trials. In each trial Gaussian, salt and pepper noises are added to the training set as described above and estimated the network performance: by plotting RI value (shown in Figures 10A,B) and by visually inspecting the map generated while presenting the input with varying noise. In the case of Gaussian noise, network shows high tolerance to the noise whose variance is <0.5. Eighty percent of the trials indicate the network fails to converge when the noise variance lies between 0.5 and 0.8 (Table 2). Similarly, in case of salt and pepper noise, network displays high tolerance to the input with pixel density ≤0.3 (i.e., 15% percentage of the image pixels were made noisy) and fails to converge when noise pixels density varies between 0.3 and 0.7 (Table 2). Thus, given network shows high tolerance (i) to the stimuli with Gaussian noise whose noise variance is <0.5 and (ii) to the stimuli with salt and pepper noise whose pixel density is <0.3. Figures 10C,D represents the percentage of pixels deviated from its preferred direction in relation to the noise density.

**Table 2:** Network robustness statistics across 20 trials.

**Noise**	**Noise density**	**% of trials**
Gaussian	0.02–0.5	1
	0.5–0.8	16
	0.8–1	3
Salt and Pepper	0.01–0.3	4
	0.3–0.7	14
	0.7–1	2

*Columns 2 and 3 together indicate the noise range at which network deviates from its clear motion selective clusters and fails to converge across trials*.

#### Varying the Bar Length

The robustness of the trained network to varying bar lengths was also investigated. The test set was created by varying bar length from 15 to 35 pixels in steps of 1 pixel. The bar length in the training set was 30 pixels. The response of the network was abstracted as robustness index. Figure 10E shows that network is highly robust to the changes in the bar length. The slight decrease in the robustness index is proportional to the difference between the bar lengths in training and test stimuli. Figure 10F shows the number of neurons deviated from its preferred direction of motion.

## Discussion

The proposed model can explain the diverse properties of the neurons present in different regions of the motion pathway. The model reproduces the motion-selective properties of cells in V1, MT, and MST. We used a hierarchical architecture consisting of neural fields to model the direction-selective cells in V1 and pattern selective cells in MT, and translational flow selective cells in MST complex. All the simulations carried out in this study, follow the same training procedure, and used the same biologically plausible asymmetric Hebb's rule to adapt the weights. The difference lies only in network size and parameter values (Table 1).

We show that the asymmetric intracortical circuitry can learn motion trajectories. In conventional symmetric Hebbian learning the pair of weights connecting a given pair of neurons, converge to the same value since symmetric Hebbian learning leads to symmetric weights. NF with symmetric weights is essentially a Hopfield network and therefore has only fixed point attractors. Such fixed point dynamics are suitable for storing static patterns as in a Hopfield network, but not for storage or generation of sequences. Even in his original paper on associative memories (Hopfield, 1982), Hopfield had suggested an asymmetric variation of the Hebb's rule for storing and generating sequences. However, such simple schemes do not perform well on large sequences and, due to the emergence of spurious states; the sequence information is quickly lost. Buchmann and Schulten (Buhmann and Schulten, 1989) have proposed a more sophisticated version of the same basic model but with extra conditions that prevent transitions to states that are not the immediate next state. Asymmetric Hebbian learning has been applied even for the problem of sequence recognition. Schultz and Reggia (Schulz and Reggia, 2004) have developed an extension of Self-Organizing Map with lateral connections trained by asymmetric Hebbian learning for recognizing phonetic sequences of words. The proposed neural field model is fashioned on similar lines as the models described above. It uses temporally asymmetric Hebbian learning to represent moving stimuli. In order to show that the temporally asymmetric is crucial to our results, we trained the network on moving oriented stimuli with both symmetric and asymmetric Hebbian learning (see Supplementary Results). The results show that the network learns to distinguish the direction of motion only when asymmetric Hebbian learning is used. It confuses between two moving bar stimuli of the same orientation and moving in opposite directions in case of symmetric Hebbian learning.

Earlier models of direction selectivity (Miikkulainen et al., 2006) and pattern selectivity by Rust et al. (2006) achieved motion sensitivity by either of two scenarios: (i) by giving the entire sequence as a stack of frames at a single time step, or (ii) a part of the stimulus is presented to the network via lagged cells. By contrast, the model proposed here has only access to the current frame. Information about the history of the stimulus is preserved in the network dynamics. When the input changes from one frame to the next the lateral interactions that were adapted to the previous frame will drive the new afferent activity and the weights updated with a new settled response will keep the memory of the history.

### The Main Findings of the Study

#### Simulation-1

The model with a single NF is trained to demonstrate direction selective properties of V1 cells. Motion selectivity is demonstrated by showing a tuned neuron response to a moving stimulus. Each neuron becomes selective to the inherent motion feature specified through a sequence of frames. Different neuron populations showed preferences to different motion directions of moving bar. Direction selectivity maps illustrated here resemble what has been observed in animals (Weliky et al., 1996). For instance, a patch of neurons with preference to a specific direction of motion will usually have a neighboring patch with preference to an opposite direction of motion (Shmuel and Grinvald, 1996). We also observed the self-organized tuned afferent weights. We revealed that the push-pull effect of lateral interactions in conjunction with weight asymmetry, develop spatiotemporal receptive fields selective for the direction of motion as found experimentally in the cortex (DeAngelis et al., 1995).

#### Simulation-2

We modeled the pattern selective responses of MT cells using the hierarchical feed-forward network, using two types of moving stimuli: (i) moving square, (ii) moving plaids.

In ***case-i***, both the NFs were trained with moving square stimuli and showed that neurons in NF1 (analogous to V1) encode the direction of local edge motion (component motion). These local motion cues are integrated and passed on to NF2 (analogous to MT) where neurons respond to the true direction of square motion. Integration of local motion cues by MT neurons was shown earlier in various experimental and modeling studies (Movshon et al., 1985; Movshon and Newsome, 1996; Simoncelli and Heeger, 1998; Pack et al., 2001; Born and Bradley, 2005). To our knowledge, ours is the first modeling study to explain the component and pattern motion selectivity using a two-dimensional object, the square.

In ***case-ii***, the first NF (analogous to V1) was trained with moving gratings and the second NF (analogous to MT) was trained with moving plaids (composed with 2 gratings). We showed that in response to moving plaid stimuli, neurons in V1 produced two activity bubbles, representing the direction of motion of plaid components (i.e., gratings). In MT single activity bubble was observed, representing the true direction of motion of plaids. These results are in accordance with earlier studies where they showed bimodal polar plots to depict responses of V1 cells and unimodal polar plots for MT cells to the moving plaid stimulus (Albright, 1984; Movshon and Newsome, 1996; Rust et al., 2006). We also plotted pattern selectivity maps and spatiotemporal receptive fields that are selective in the direction of pattern motion.

#### Simulation-3

In this study we simulated a network with two NFs, using more complex stimuli: RDS sequences that follow translational trajectories, to simulate the translational flow selective properties of the neurons at MST. A set of 25 random dot configurations were created and each move in 4 directions to create 100 sequences. NF1, NF2, and perceptron were trained one after the other with sequences created from 20 configurations. Remaining 5 sequences considered as a test set. Now the trained network was presented with the training set. It showed that the NF2 neurons can encode the coherent motion direction of the dots, independent of the dot configuration. When the test set was presented, it showed that the network can extract the direction of motion of the dots in unseen sequences with an accuracy of 90%. Thus, the proposed network can be generalized to extract the motion direction in translational flow sequences. Unlike in earlier simulations in this simulation, we considered RDS moving in 4 directions. Also, the image size is reduced to 32 × 32 pixels. This reduction is done to reduce the computational expense.

### Future Studies

In the third study, we proposed and explored network for translational flow selectivity using translational random dot sequences. There are other variants of optic flow, such as radial flow (expansion/contraction) and circular flow (clockwise and anticlockwise rotation). The brain region that is selective to the translational flow is different from the region that is selective for radial and rotational flow (Morrone et al., 2000). In future studies, we would like to explore and simulate the neurons (as NF3) that are selective for radial and rotational flow. Also, we would like to simulate the more biologically plausible models on real-world visual motion inputs. For example, instead of NFs consisting of sigmoidal neurons, we would like to explore more realistic neuron models like the FitzHugh-Nagumo neuron which is likely to present richer dynamics more suitable for motion processing.

## Methods

### Moving Bar Stimuli

Rectangular white bars of length 30 pixels and width 2 pixels were oriented in the orthogonal direction of motion were made to move on black background of size 64 × 64 pixels. The bar moving from one end to other in a specific direction creates a single sequence. A set of 8 such sequences were created to train the network by moving the bar in 90, 135, 180, 225, 270, 315, 0, and 45°. Each video sequence is made up of 8 frames with bar displacement (step size) of 7.8 pixels. Single neuron experiments reported that most of the V1 direction-selective neurons are highly selective if stimulus motion direction is perpendicular to its orientation (Albright, 1984).

### Moving Gratings and Plaids Stimuli

Moving plaid patterns were generated by superimposing two orthogonal sinusoidal gratings, having the same spatial frequency and moving at the same speed. Two orthogonal gratings with the same spatial frequency have a strong tendency to cohere (Adelson and Movshon, 1982). So first we generated drifting gratings that move orthogonally to its spatial orientation. A single point at which the loci of grating motions intersect will give the plaid motion (Adelson and Movshon, 1982), so we combined gratings separated by 90° to generate plaids. Gratings and plaids are allowed to move in 8 directions: 0, 45, 90, 135, 180, 225, 270, and 315°. For instance, the plaid moving in 45° is generated by the perceptual coherence of two gratings moving in 0 and 90°. The training set was generated with video sequences of moving gratings and moving plaids. Each moving grating sequence is composed of 10 frames with a frame size of 64 × 64. The spatial frequency of the grating is set to 5 pixels.

### Moving Square Stimuli

The training set is made up of 8 fixed length sequences with 5 frames each. Each moving stimulus consists of White Square of size 24 × 24 pixels, moving through the origin over a black background of size 64 × 64 pixels. The white square was moved in 8 possible directions: 0, 45, 90, 135, 180, 225, 270, and 315° from 8 different starting positions.

### RDS-Translation Stimuli

Random dot stimuli were generated by positioning 16 white dots (actually they are tiny squares and assuming them as dots for simplicity) of size 2 × 2 pixels randomly upon a black square grid of size 32 × 32 pixels with a constraint that each 8 × 8 window of black background can accommodate only one dot. A set of 25 such dot configurations were created and each configuration is moved (displacing X, Y coordinates one location ahead at a time) in 4 directions (θ): 0, 90, 180, 270°. If the dot exceeds the square boundary of the frame, it was wrapped around to reappear on the opposite side of the frame; thus the dot density across the frames was kept constant. Hundred translational random dot sequences were produced with 5 frames each. Out of 100, 80 sequences were used as training set, and the remaining 20 sequences were used as a test set. All the above inputs were programmed in MATLAB.

### Perceptron

Single layer multiclass perceptron with input and output layers were used to classify the response of the neural field network and assess its performance. The number of units in the perceptron input layer is equal to a number of neurons in the NF layer from which perceptron receives input. The number of units in the output layer is equal to the number of classes. Thus, perceptron network size is different for different simulations. The equations that govern learning are:

$O_{i}=\;g\left(\underset{j}{\sum }{W_{ji}I}_{j}+b\right)$

*E* = *y*_*i*_ − *O*_*i*_

Δ*W*_*j*_ = α*I*_*j*_*E*

Δ*b*_*j*_ = α*E*

where g = Sigmoid function, *y*_*i*_ be the correct output, *O*_*i*_ be the actual output, *E* is the error, α is the learning rate whose value is 0.1 in the simulation.

## Author Contributions

AG performed designing and coding the model, input generation, running and analyzing simulations, and manuscript preparation. VC and KS performed designing the model and manuscript preparation.

### Conflict of Interest Statement

The authors declare that the research was conducted in the absence of any commercial or financial relationships that could be construed as a potential conflict of interest.

## Abbreviations

NF: Neural Field
V1: Primary Visual Cortex
MT: Middle Temporal Area
MST: Medial Superior Temporal Area
RDS: Random Dot Stimulus
RF: Receptive Field.
