# Supplementary material for: A Model of Motion Processing in the Visual Cortex Using Neural Field With Asymmetric Hebbian Learning
Source: Front Neurosci. 2019 Feb 12;13:67. doi: 10.3389/fnins.2019.00067 (PMC6380226; doi:10.3389/fnins.2019.00067)
Supplement: Supplementary file 1 [file Table_1.docx]

**Supplementary Results**

**Manuscript title**: A model of motion processing in the visual cortex using neural field with asymmetric Hebbian learning
**Manuscript ID**: 428656 
**Authors:**  Anila Gundavarapu, Srinivasa Chakravarthy, Karthik Soman

**No. of Figures: 2**

**1. Asymmetric Hebbian Learning Vs Symmetric Hebbian Learning**

Temporally asymmetric Hebbian learning was a mechanism that support sequence processing ([Schulz and Reggia 2004](#_ENREF_4)). The experimental evidence showing that changes in biological synaptic efficacy in the cortex ([Bi and Poo 1998](#_ENREF_1); [Bi and Poo 2001](#_ENREF_2)) are sometimes due to temporally asymmetric Hebbian learning: a synapse is strengthened (long-term Potentiation, LTP), if presynaptic action potentials precede excitatory post synaptic potentials by typically 20 to 50 ms, and weakened (long-term depression, LTD) if the time course is reversed ([Schulz and Reggia 2004](#_ENREF_4)).

Given two NF neurons *i* and *j*. In symmetric Hebbian, the connection strength *W_ij_* to *i* from *j* at time *t* is increased proportional to the activity of *j*, times the activity of *i*. The other connection strength *W_ji_* to *j* from *i* is also approaches to the same value, during training, irrespective of the initial random connection strengths. However in the asymmetric rule described here, the increase in the connection strength *W_ij_* is proportional to the activity of *j* at previous time step, times $\max\left( 0, \eta_{i}\left( t \right)-\eta_{i}\left( t-1 \right) \right)$, increase in the activity of *i* relative to the previous time step. This makes the connections between given two neurons *i*, *j* asymmetric. In general the eqn (4) (given in Manuscript) capture the notation of cause and effect across time ([Schulz and Reggia 2004](#_ENREF_4)). The connection *W_ij_* is strengthened if *j*’s activity at *(t-1)* contributes to an increase in the activation of *i*. The previous results ([Roberts 1999](#_ENREF_3)) using temporally asymmetric Hebbian reported that the overall change in the synaptic strength is proportional to the rate of change in the post synaptic activity ([Schulz and Reggia 2004](#_ENREF_4)).

Afferent activity is a time variant. For each frame in the sequence this activity varies. The lateral connections adapted using asymmetric Hebbian, learn the correlations between afferent activities and become selective to the time invariant input feature, direction of motion in this case. Intuitively the lateral connections between the neurons in NF capture the temporal relations in the input and form unique spatial representation for each of the input. To prove that the lateral connections become selective to the time variant input features, we trained single NF network using symmetric Hebbian on moving bar stimuli by using the same parameter set that was used for asymmetric weight connections. The response of the trained network is shown in the Fig. S2. We observed 4 spatially distinct activation patterns on NF in response to 8 inputs. The sequences with same orientation were transformed into same spatial representation after training. This suggests that asymmetrically learned lateral connections are the major cause of the unique spatial representations of the input space. To quantify it, we measured the distance between two final activation patterns of moving bar inputs that have same orientation and opposite motion directions. We used L1-Norm ([Schulz and Reggia 2004](#_ENREF_4)) as a distance measure. Let *Z^1^, Z^2^* be the two final activation patterns of bar with same orientation moving in opposite directions, the distance between them is measured as:

$d(Z^{1} - Z^{2})=\left\| Z^{1}-Z^{2} \right\|1= \sum_{n=1}^{N} \left| Z_{n}^{1}-Z_{n}^{2} \right|$

The distance measure is applied to 4 pairs: ( 0 ̊ , 180 ̊ ),( 45 ̊ , 225 ̊ ),( 90 ̊ , 270 ̊ ),( 135 ̊ , 315 ̊) in both models (model trained with symmetric Hebbian and the model trained with asymmetric Hebbian); the result is shown in a bar graph Fig S2D. The model trained using asymmetric Hebbian is separating input pairs into unique spatial representations and shows high distance value. The model trained using symmetric Hebbian confused between the sequences of each pair and shows low distance value.

**References:**

Bi, G.-q. and M.-m. Poo (1998). "Synaptic modifications in cultured hippocampal neurons: dependence on spike timing, synaptic strength, and postsynaptic cell type." Journal of Neuroscience **18**(24): 10464-10472.

Bi, G.-q. and M.-m. Poo (2001). "Synaptic modification by correlated activity: Hebb's postulate revisited." Annual review of neuroscience **24**(1): 139-166.

Roberts, P. D. (1999). "Computational consequences of temporally asymmetric learning rules: I. Differential Hebbian learning." Journal of Computational Neuroscience **7**(3): 235-246.

Schulz, R. and J. A. Reggia (2004). "Temporally asymmetric learning supports sequence processing in multi-winner self-organizing maps." Neural Computation **16**(3): 535-561.

**Fig S1: RFs developed on NF1:** spatio temporal receptive fields developed as result of training NF1 (20 x 20 units) with moving bar stimuli. Neighboring neurons shows preferences to similar inputs and trained RFs are arrange in a topographic fashion. Four big cluters of neurons can be seen which indicate neurons in each cluster have similar preference to the orientation of bar.

**Fig S2: Symmetric Vs Asymmetric network response to the moving bar stimuli:** (A) column 1 represents the first frame of the moving bar stimuli; coulumn 2 and 3 represents the the responses of two trained networks. Network trained using asymmetric lateral weight connections transformed each input pattern into *unique spatial representation* on the map as shown in B, whereas the network with symmetric weight connections transformed inputs with same orientations into *similar spatial representation* on the map as shown in C. Distance between these similar spatial representations were computed and compared it with that of distances produced by asymmetric network as shown in D. Less distance between spatial patterns in case of symmetric network indicates that the lateral asymmetric weight connections are important for learning time invariant feature of the input, which is direction of motion in this case.
