# Supplementary figures and images for: A Model of Motion Processing in the Visual Cortex Using Neural Field With Asymmetric Hebbian Learning
Source: Front Neurosci. 2019 Feb 12;13:67. doi: 10.3389/fnins.2019.00067 (PMC6380226; doi:10.3389/fnins.2019.00067)

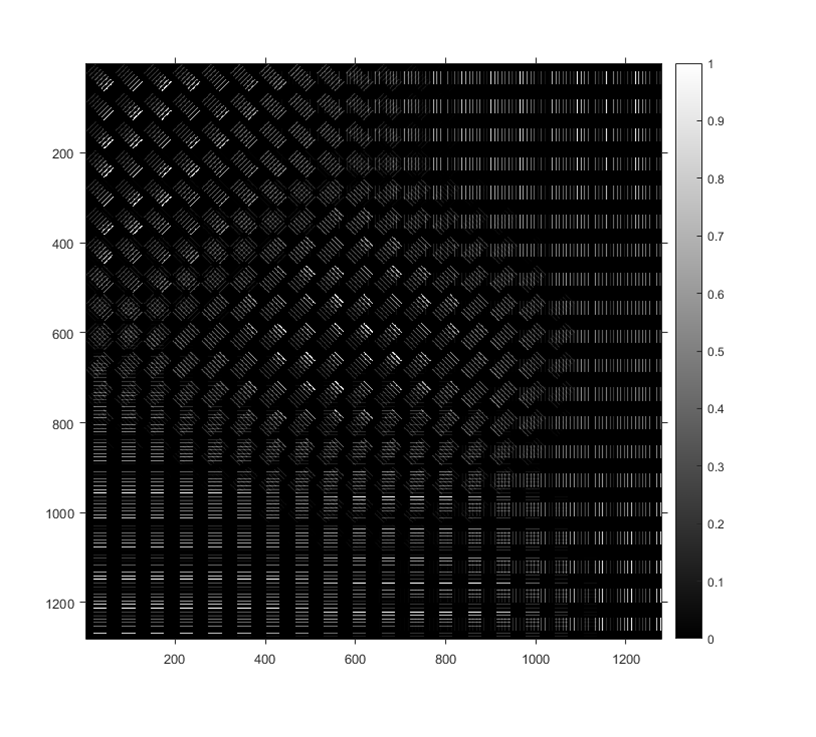

Supplement: Supplementary file 2 [file Image_1.TIF]

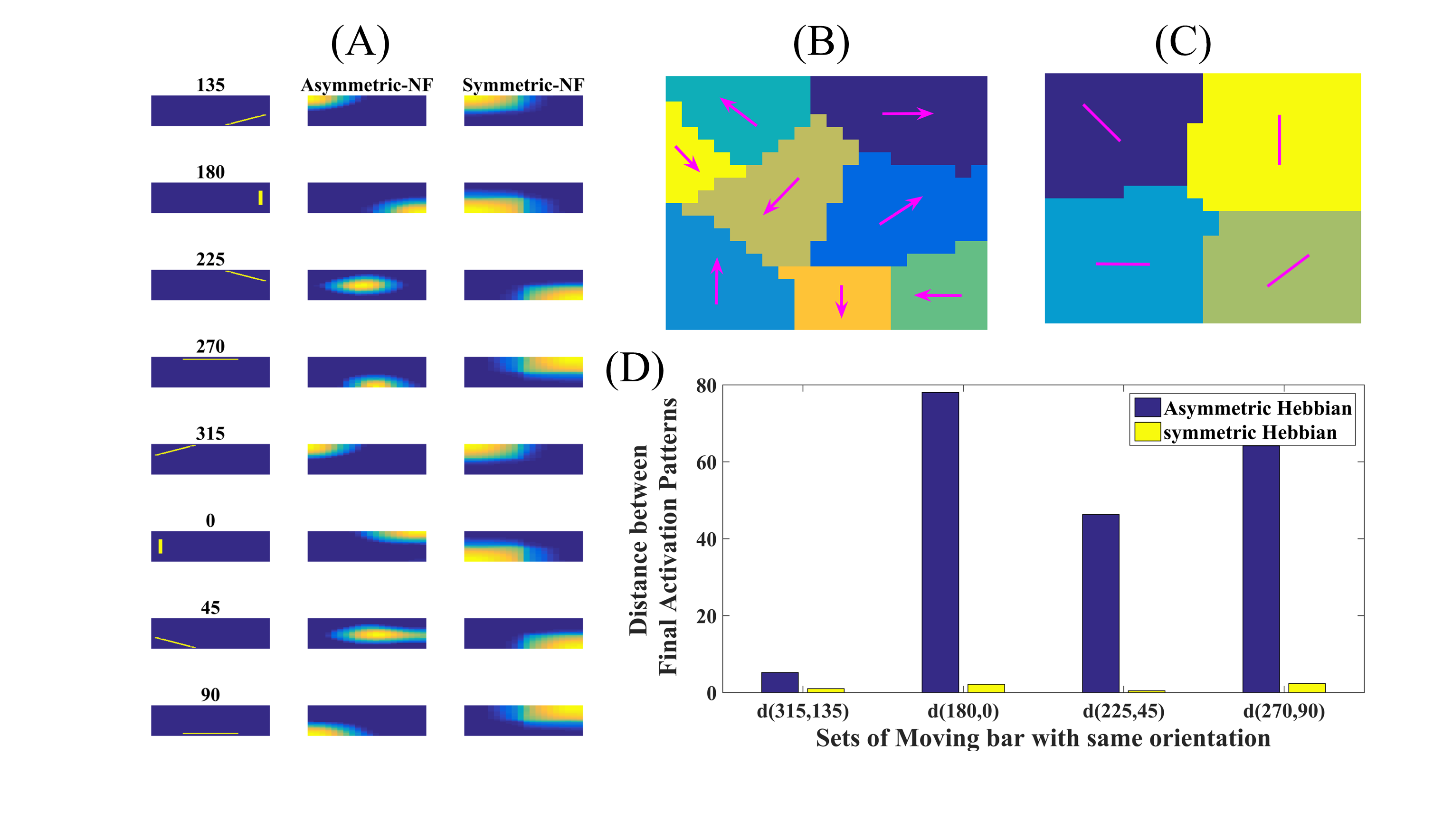

Supplement: Supplementary file 3 [file Image_2.TIF]
